# Developing a set of strong intronic promoters for robust metabolic engineering in oleaginous *Rhodotorula* (*Rhodosporidium*) yeast species

**DOI:** 10.1186/s12934-016-0600-x

**Published:** 2016-11-25

**Authors:** Yanbin Liu, Sihui Amy Yap, Chong Mei John Koh, Lianghui Ji

**Affiliations:** 1 Biomaterials and Biocatalysts Group, Temasek Life Sciences Laboratory, National University of Singapore, 1 Research Link, Singapore, 117604 Singapore; 2 School of Biological Sciences, Nanyang Technological University, 60 Nanyang Drive, Singapore, 637551 Singapore

**Keywords:** *Rhodosporidium/Rhodotorula*, Metabolic engineering, Oleaginous yeast, Promoter, Lipid

## Abstract

**Background:**

Red yeast species in the *Rhodotorula/Rhodosporidium* genus are outstanding producers of triacylglyceride and cell biomass. Metabolic engineering is expected to further enhance the productivity and versatility of these hosts for the production of biobased chemicals and fuels. Promoters with strong activity during oil-accumulation stage are critical tools for metabolic engineering of these oleaginous yeasts.

**Results:**

The upstream DNA sequences of 6 genes involved in lipid biosynthesis or accumulation in *Rhodotorula toruloides* were studied by luciferase reporter assay. The promoter of perilipin/lipid droplet protein 1 gene (*LDP1*) displayed much stronger activity (4–11 folds) than that of glyceraldehyde-3-phosphate dehydrogenase gene (*GPD1*), one of the strongest promoters known in yeasts. Depending on the stage of cultivation, promoter of acetyl-CoA carboxylase gene (*ACC1*) and fatty acid synthase β subunit gene (*FAS1*) exhibited intermediate strength, displaying 50–160 and 20–90% levels of *GPD1* promoter, respectively. Interestingly, introns significantly modulated promoter strength at high frequency. The incorporation of intron 1 and 2 of *LDP1* (*LDP1in* promoter) enhanced its promoter activity by 1.6–3.0 folds. Similarly, the strength of *ACC1* promoter was enhanced by 1.5–3.2 folds if containing intron 1. The intron 1 sequences of *ACL1* and *FAS1* also played significant regulatory roles. When driven by the intronic promoters of *ACC1* and *LDP1* (*ACC1in* and *LDP1in* promoter, respectively), the reporter gene expression were up-regulated by nitrogen starvation, independent of de novo oil biosynthesis and accumulation. As a proof of principle, overexpression of the endogenous acyl-CoA-dependent diacylglycerol acyltransferase 1 gene (*DGA1*) by *LDP1in* promoter was significantly more efficient than *GPD1* promoter in enhancing lipid accumulation.

**Conclusion:**

Intronic sequences play an important role in regulating gene expression in *R. toruloides.* Three intronic promoters, *LDP1in*, *ACC1in* and *FAS1in*, are excellent promoters for metabolic engineering in the oleaginous and carotenogenic yeast, *R. toruloides.*

**Electronic supplementary material:**

The online version of this article (doi:10.1186/s12934-016-0600-x) contains supplementary material, which is available to authorized users.

## Background

Red yeast species in the *Rhodosporidium* (teleomorph) genus, which was recently revised as genus *Rhodotorula* (anamorphic) regarding to the implementation of “One Fungus = One Name” nomenclatural principle [1], are outstanding producers of lipids and carotenoids [2, 3]. More than 100 g/L of dry biomass with over 60% neutral lipids (triacylglycerol, TAG) content can be produced within a week when glucose was used as the carbon source [4–6]. To take advantage of their high metabolic flux and cell mass productivity, a number of laboratories have been engaged in establishing them as new platforms for synthetic biology and metabolic engineering. To date, several genetic manipulation tools have been reported, such as high efficiency transformation via *Agrobacterium tumefaceins*-mediated transformation (ATMT), high efficiency gene deletion, and constitutive and inducible promoter toolbox [7–12]. As oil producers, strong and robust promoters that function during lipid accumulation stage will be particularly useful.

The production of long chain acyl-CoA, triacylglycerol (TAG) and lipid bodies are the 3 major lipogenesis steps, and genes involved in these processes are the likely source of strong promoters in oleaginous yeasts. Acetyl-CoA carboxylase (Acc1) catalyzes the biotin-dependent carboxylation of acetyl-CoA to form malony-CoA, the first committed and rate-limiting step in fatty acid biosynthesis [13]. Proteomic studies showed that it is a highly abundant protein during lipid accumulation phase [5]. Likewise, the α and β subunits of fatty acid synthase (Fas2 and Fas1, respectively), fatty acid transporter (Fat1), ATP:citrate lyase (Acl1) and urea carboxylase/allophanate hydrolase (Dur1/2) were also found in high abundance during lipid accumulation phase [14, 15]. Other known abundant targets are the perilipin, adipophilin and tail-interacting (PAT) family proteins, which serve as dynamic scaffolds regulating the formation, growth and degradation of lipid bodies [15–19].

Eukaryotic genes are often interrupted by spliceosomal introns, which vary greatly among different species in either density (number of introns per gene) or length [20]. Although introns are often perceived as junk DNA gained during genome evolution [21], some introns are known to regulate gene transcription and this effect is known as intron-mediated enhancement (IME) [22]. The concept of IME was initially reported in plant [22] and subsequently observed in other organisms such as flies [23] and fungi [24, 25]. To our knowledge, two cases of IME have been reported in oleaginous yeasts where the 5′ introns significantly enhanced gene expression: one in fructose 1,6-bisphosphate aldolase gene (*FBA1*) of *Yarrowia lipolytica* and the other in d-amino acid oxidase gene (*DAO1*) of *R. toruloides* [12, 26]. Despite Acc1 being the most abundant protein in *R. toruloides* [5], the 1.5-kb upstream DNA sequence of *ACC1* (−1501 to −1 from the translational start site) showed little promoter activity (our unpublished data). In addition, the high intron density (an average of 6 introns per gene) [14] and strong enhancing effect of the *DAO1* introns [12] suggested the global regulatory roles of introns in *R. toruloides*.

Here, we report the cloning and molecular characterization of 6 promoters from *R. toruloides* and demonstrate their applications in metabolic engineering.

## Results

### Characterization of genes involved in lipid accumulation

**Fig. 1:**
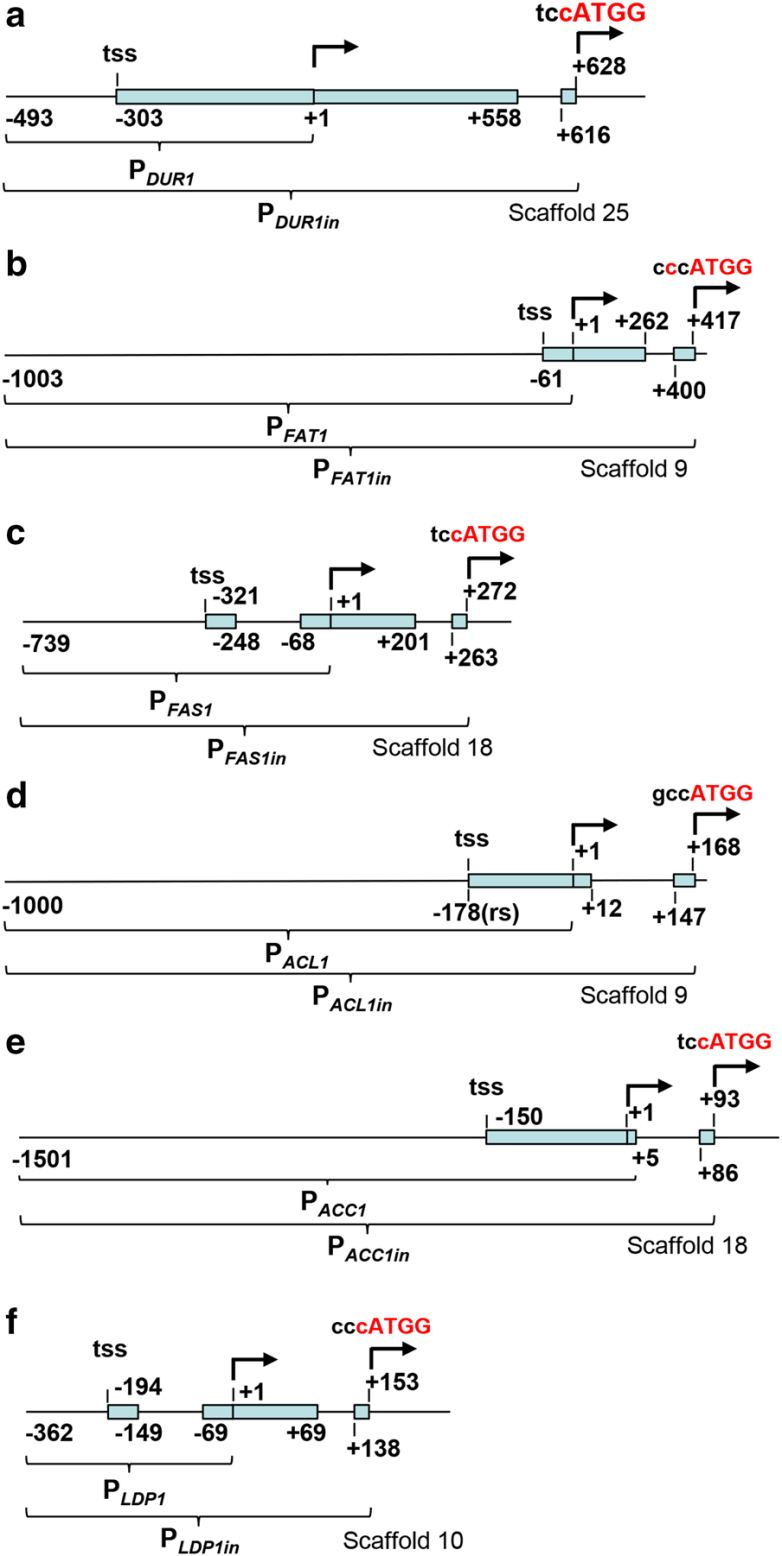
Schematic diagrams of promoters. **a** *DUR1* and *DUR1in* promoters. **b** *FAT1* and *FAT1in* promoter. **c** *FAS1* and *FAS1in* promoters. **d** *ACL1* and *ACL1in* promoters. **e** *ACC1* and *ACC1in* promoters. **f** *LDP1* and *LDP1in* promoters. tss represents the transcription start site,* blue bars* represent exons. Translational starts (ATG) in intronic promoters are indicated. Nucleotides in* red letters* indicate modifications from the genome sequences. The scaffold number is based on the genome sequence of *R. glutinis* ATCC 204091 [28]

Genomic sequences for acetyl-CoA carboxylase gene (*ACC1*), ATP:citrate lyase gene (*ACL1*), β subunit of fatty acid synthetase gene (*FAS1*), fatty acid transporter gene (*FAT1*) and urea amidolyase gene (*DUR1*) were identified by BLAST search against the public database as well as in-house EST and genome database of *R. toruloides* strains [27, 28]. The amino acid sequences of known orthologous enzymes from *Saccharomyces cerevisiae* or *Y. lipolytica* were used as queries (Table 1). The perilipin encoding gene of *R. toruloides* NP11 strain (lipid droplet protein 1 gene, *LDP1*) [15] was used to search for its counterpart in *R. toruloides* ATCC 10657. The putative homolog of *ACC1*, *ACL1*, *FAS1*, *FAT1*, *DUR1* and *LDP1* was found located in the genome sequencing scaffold No.18, 9, 18, 9, 25 and 10 of *R. glutinis* ATCC 204091, respectively (Table 1). Analysis by 5′ RACE and transcriptomics showed that the cDNA of *ACC1*, *ACL1*, *FAS1*, *FAT1*, *DUR1* and *LDP1* contains a 5′ untranslated region (5′UTR) of 150, 179, 142, 61, 303 and 194 nt in length, respectively (Table 1). Notably, the first intron was found to be located within the 5′UTR of both *FAS1* and *LDP1* (Fig. 1). The detailed structures and sequences of these genes are summarized in Table 1 and Additional file 1, respectively.

**Table 1:** Gene annotations

Gene	CDS length (nt)	Scaffold No.	5′UTR (nt)	3′UTR (nt)	Exon	Protein (aa)	Query^a^
*ACC1*	7347	18	150	187^b^	11	2232	YNR016C
*ACL1*	4417	9	178^b^	216	10	1157	YALI0E34793g
*FAS1*	9628	18	142^bc^	101^b^	16	2928	YKL182W
*FAT1*	2860	9	61^b^	105^b^	14	639	YBR041W
*DUR1*	4446	25	303^b^	109^b^	12	1239	YBR208C
*LDP1*	1256	10	115^bc^	230^b^	7	261	RHTO-05627

^a^ Genbank numbers used for BLAST search and gene annotation

^b^ Predicted by transcriptomic results

^c^ Containing the first intron within 5′UTR

### Analysis of promoter activity by luciferase reporter assay

Upstream DNA sequences of the above mentioned genes were amplified by PCR in two versions, with or without intronic sequence (Fig. 1), and fused to the codon-optimized luciferase reporter gene Rt*LUC2* (GenBank accession number KR258785) [12] in the binary vector pKCL2. pKCL2 allows site-specific integration of reporter cassettes at the CAR2 locus (phytoene synthase/carotene cyclase gene), which eliminates position effects caused by ectopic insertion of T-DNA into the chromosomes [8, 12]. The names of intronic promoters were affixed with “*in*” to differentiate the promoters analyzed.

**Fig. 2:**
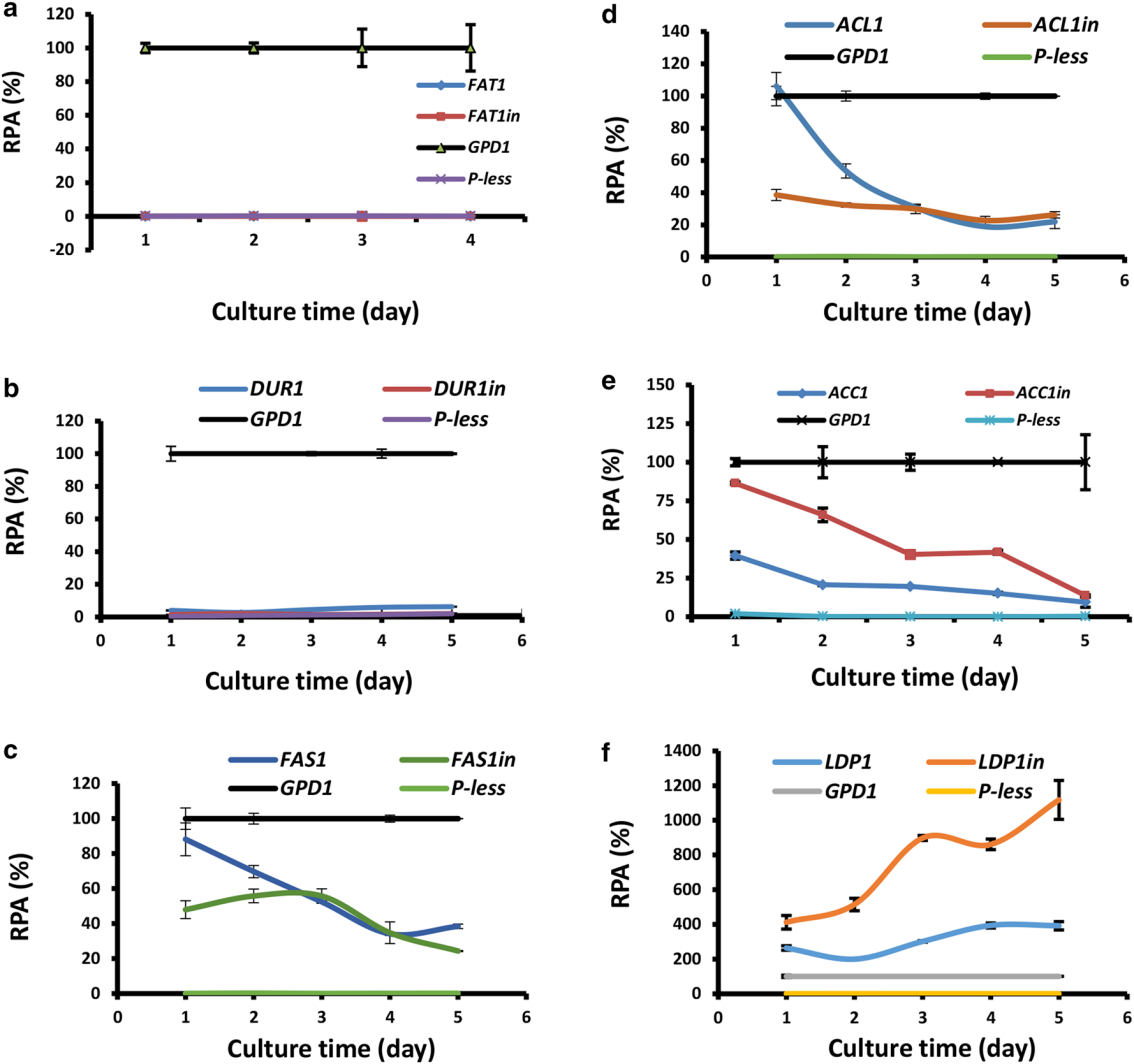
Time-course studies of promoter strength by luciferase gene reporter assay. **a** *DUR*1 and *DUR1in* promoter. **b** *FAT1* and *FAT1in* promoter. **c** *FAS1* and *FAS1in* promoter. **d** *ACL1* and *ACL1in* promoter. **e** *ACC1* and *ACC1in* promoter. **f** *LDP1* and *LDP1in* promoter. Cells were cultured in MinRL3 medium at 30 °C. Results were derived from three biological replicates and* error bars* indicate standard deviation. *RPA* relative promoter activity normalized against that of *GPD1* promoter

As reported, *DUR1* and *FAT1* were highly transcribed during lipogenic phase [14]. However, luciferase reporter assay revealed that none of *DUR1*, *DUR1in*, *FAT1* and *FAT1in* promoters (Fig. 1a, b) displayed detectable activity throughout the cell culture (Fig. 2a, b). The presence of introns in *FAS1* and *ACL1* promoters weakened promoter activities at the initial stages of cell culture (day 1 and 2) (Fig. 2c, d). However, these repressive effects disappeared after day 3 when nitrogen levels became limited and lipid accumulation began to accelerate (Fig. 2c, d). Thus, the FAS1in and ACL1in promoters (Fig. 1c, d) should be useful under circumstances where strong expression have undesirable effects at the early stages of cell culture. Otherwise, the intronless *FAS1* and *ACL1* promoters would be preferred for gene expression. Unlike the intronless *ACC1* promoter (−1501 to −1, Fig. 1e), the *ACC1in* promoter (−1501 to +93) yielded significantly higher luciferase activity throughout the time course (Fig. 2e).

Suprisingly, *LDP1* promoter (−362 to −1, Fig. 1f) exhibited two- to fourfold higher activity than the *GPD1* promoter, one of the strongest promoters known in fungi (Fig. 2f). Moreover, the intronic LDP1in promoter (−362 to +155, Fig. 1f) further enhanced the promoter activity, reaching up to 11 times that of GPD1 promoter (Fig. 2f). Taken together, promoters of *FAS1*, *ACC1* and *LDP1* are strong candidates for metabolic engineering in *R. toruloides* and related fungal species.

**Fig. 3:**
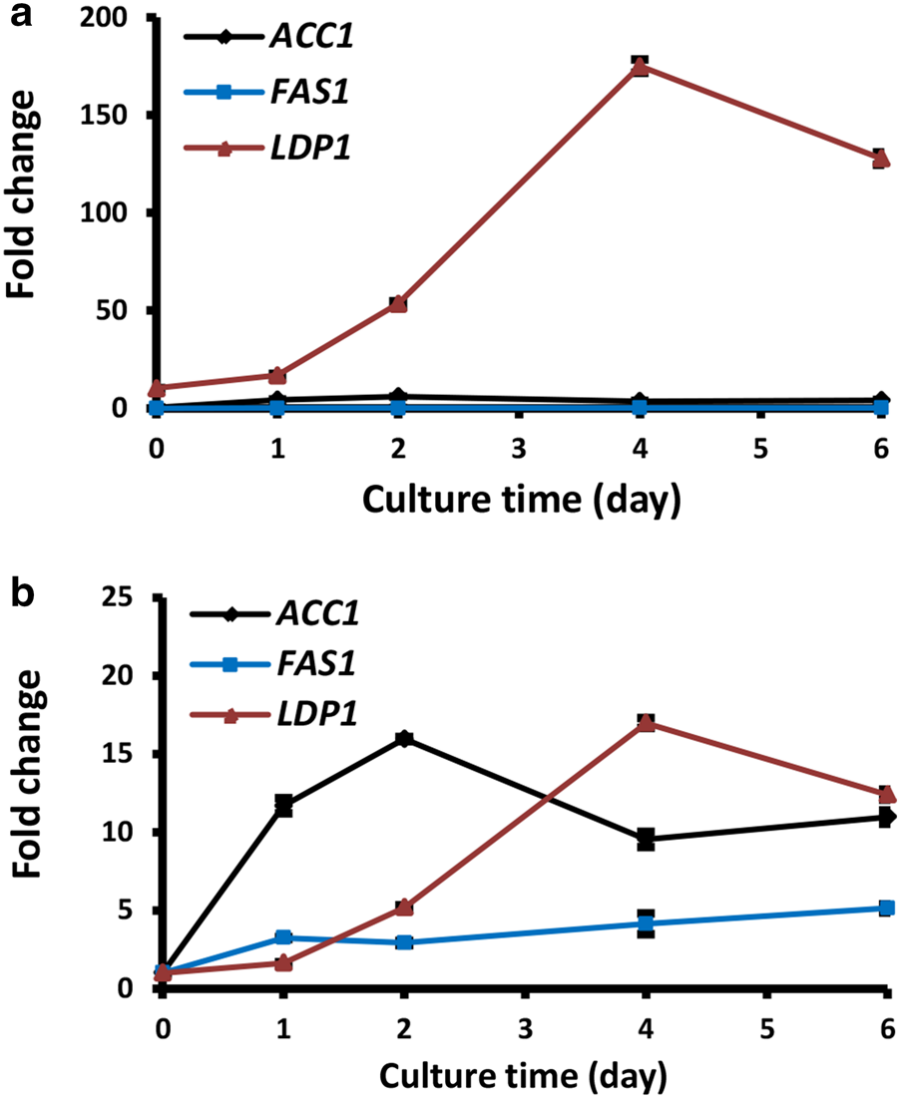
qRT-PCR analysis of mRNA levels. **a** Relative mRNA levels of *ACC1*, *FAS1* and *LDP1*. Calculation of mRNA levels were done using 2^−ΔCt^ method by normalizing against actin gene *ACT1* (reference). **b** Dynamic changes of mRNA levels of *ACC1*, *FAS1* and *LDP1*. Fold change of mRNA level was normalized against its own mRNA level at day 0. WT strain was cultured in MinRL3 medium for 6 days at 30 °C. Results were derived from three biological replicates and *error bars* indicate standard deviation

### Transcription of *ACC1*, *FAS1* and *LDP1* mRNAs during lipid production

Gene expression of *ACC1*, *FAS1* and *LDP1* were investigated by qRT-PCR analysis, which was done by normalizing against the transcripts of actin gene (*ACT1*) using cycle threshold (Ct) method (2^−ΔCt^ calculation method). In good agreement with the luciferase reporter assays, mRNA levels of *LDP1* were much higher than those of *ACC1* and *FAS1* throughout the 6-day culture period (Fig. 3a). To show the dynamic changes in gene expression, the relative mRNA levels of *ACC1*, *FAS1* and *LDP1* at different time points were compared using 2^−ΔΔCt^ calculation method, where each mRNA level at day 0 was set as 1. Overall, all 3 genes showed upward trends in transcription over the 6-day culture period, although the mRNA levels of *ACC1* and *LDP1* peaked earlier at day 2 and 4, respectively (Fig. 3b).

### Performance of promoters in a lipid-less genetic background

**Fig. 4:**
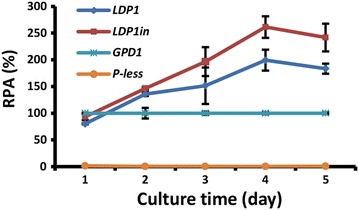
Promoter strength in lipid production-deficient mutant *dlad*. The *dlad* strain is mutant with targeted deletion of four diacylglycerol acyltransferase genes (our unpublished data). All reporter mutants were cultured in MinRL3 for 5 days and luciferase reporter assays were performed daily. Results were derived from three biological replicates and* error bars* indicate standard deviation

To evaluate the performance of the above promoters in a non-oil accumulating genetic background, reporter constructs for *LDP1* and *LDP1in* were transformed into a *R. toruloides* mutant named *dlad*, in which genes encoding 4 essential acyltransferase in lipid biosynthesis, acyl-CoA-dependent diacylglycerol acyltransferase (*DGA1*), phospholipid:diacylglycerol acyltransferase (*LRO1*), acyl-CoA:sterol acyltransferase (*ARE1*) and soluble diacylglycerol acyltransferase (*DGA3*), were sequentially disrupted (our unpublished data). This quadruple disruption mutant contains only 8.5% lipids of wild-type strain (WT) (our unpublished data). Similar to the situation in WT, both *LDP1* and *LDP1in* promoters displayed significantly higher activity than the *GPD1* promoter in *dlad* mutant. Moreover, the *LDP1in* promoter was more active than the *LDP1* promoter (Fig. 4) although the magnitude of enhancement appeared to be reduced (compare Figs. 2f, 4). These suggest that the lipogenic promoters such as *LDP1* and *LDP1in* are largely regulated by nutrient levels independent of de novo TAG biosynthesis.

### Metabolic engineering of lipid production using different promoters

**Fig. 5:**
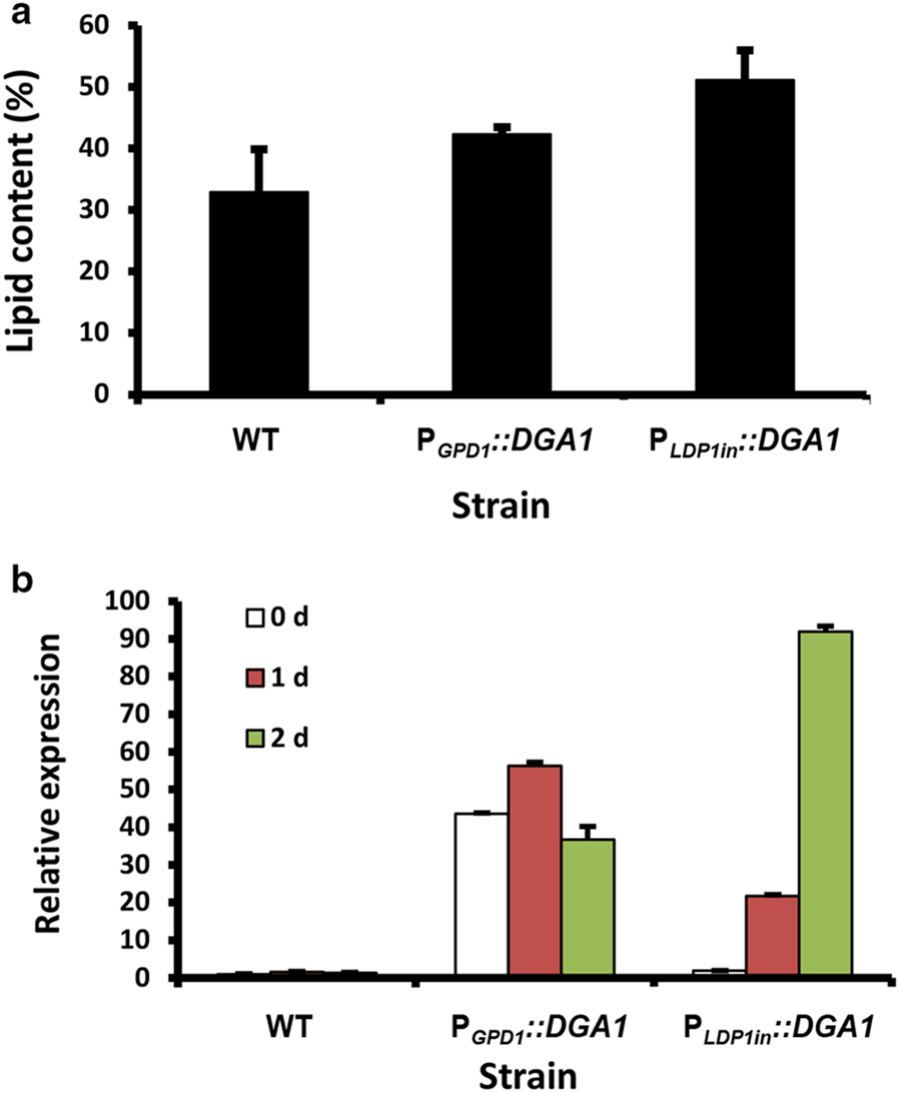
Enhancement of lipid content by *DGA1* overexpression. **a** Quantification of lipid content as gram lipids per gram dry cell weight (%). **b** qRT-PCR analysis of *DGA1* expression. WT, P_*GPD1*_ *::DGA1* and P_*LDP1in*_ *::DGA1* strains were cultured in GJ2013 medium for 3 days at 30 °C. Results were derived from three biological replicates and* error bars* indicate standard deviation

Overexpression of Dga1, the key enzyme of TAG biosynthesis, has been demonstrated to significantly improve lipid yields in other oleaginous yeast like *Y. lipolytica* [13]. As a principle of concept, to demonstrate the application of the strong promoters characterized in this study, we compared the lipid production levels by overexpressing *DGA1* using either *GPD1* or *LDP1in* promoter (strain P_*GPD1*_::*DGA1* and P_*LDP1in*_::*DGA1*, respectively). Results showed that overexpression of *DGA1* with either promoters significantly improved lipid content (Fig. 5a). More importantly, lipid content accumulated in the strain P_*LDP1in*_::*DGA1* was on average 21% higher than that in the strain P_*GPD1*_::*DGA1* and 55% higher than that of WT strain (Fig. 5a). qRT-PCR analysis was used to identify the gene expression of *DGA1* in the above three strains. When compared to WT strain on day 2, the use of *GPD1* and *LDP1in* promoter resulted in 26- and 66-fold increase in the mRNA levels of *DGA1*, respectively (Fig. 5b). These data suggest that the promoters developed in this study, particularly the *LDP1in* promoter, are superior tools for metabolic engineering in oleaginous yeasts.

## Discussion

Lipid biosynthesis is the dominant bioactivity in oleaginous yeasts. Therefore, promoters of genes involved in various steps of the pathway are particularly useful. Such examples include *GPD1*, *PGK1* and *PGI1* promoters as reported previously [7, 11]. Here, we analyzed the promoters of 6 genes involved in either fatty acid biosynthesis, TAG biosynthesis or lipid body formation. Through luciferase reporter assay, the application of intronic promoters derived from *ACC1*, *FAS1* and *LDP1* genes were firmly established.

Promoters are generally known to be located upstream of the transcriptional start site (TSS) and composed of a core promoter domain and several regulatory domains [29]. Although *cis*-acting elements may be located 50 or more kilobases proximal to the TSS, most eukaryotic promoters used to date are within 1 kb upstream from the TSS, including those reported promoters from *R. toruloides* [7, 12]. However, increasing evidences have demonstrated that *cis*-acting elements may be located downstream of the TSS, in the 5′UTR, within an intron, or even downstream from the last exon of a gene [30]. The inclusiveness of introns in certain genes may improve their transcriptional and translational outputs [31]. Regulatory elements in the first intron within the 5′UTR and coding region have been found in higher eukaryotes elsewhere [32–36]. Although IME has been known for a long time, mechanistic studies are still lagging. It was recently proposed that introns may create a region of localized accessible chromatin to increase transcriptional initiation [37]. The introns characterized in this report with diverse roles in regulating gene expression should serve as good samples for the mechanistic studies of IME.

In this study, we found that the expression of 5 out of 6 genes were significantly regulated by introns: with strong enhancing effect on *ACC1* and *LDP1* genes and repressing effect on *ACL1*, *FAS1* and *DUR1* genes (Fig. 2). In fact, intron 1 of *GPD1* gene (+3 to +131) also contains a repressing element (Additional file 2). These results, together with the previous report of *DAO1* gene [12], showed that the involvement of introns in transcriptional control is a universal phenomenon in *R. toruloides*. Further studies in this area may facilitate the development of a more robust and efficient synthetic platform based on oleaginous red yeasts.

## Conclusions

Intronic promoters of *ACC1*, *FAS1* and *LDP1* are superior tools for gene expression, metabolic engineering and synthetic biology in *R. toruloides*. Similar promoters may also be functional in other *Pucciniomycotina* species.

## Methods

### Strains, media and culture conditions

*Rhodotorula toruloides* strain ATCC 10657 was purchased from ATCC (Manassas, VA, USA). *R. toruloides* nonhomologous end joining-deficient mutant strain Δku70e [8] was considered as the wild-type strain. *R. toruloides* quadruple disruption mutant *dlad* (*Δdga1Δlro1Δare1Δdga3*) was generated by serial deletion of the four diacylglycerol acyltransferase genes, *DGA1*, *LRO1*, *ARE1* and *DGA3*, in the host Δku70e through homologous recombination, in which the selectable marker cassette was recycled by activating Cre/*loxP* system [8]. *A. tumefaciens* strain AGL1 [38] and *Escherichia coli* XL1-BLUE were used for routine molecular cloning procedures.

*Rhodotorula toruloides* was cultured at 28 °C in YPD broth (1% yeast extract, 2% peptone, 2% glucose) or on solid potato-dextrose agar (PDA). *A. tumefaciens* was grown at 28 °C in either liquid or solid 2YT medium (1.6% tryptone, 1% yeast extract, 0.5% NaCl). *E. coli* XL1-Blue was cultured in Luria–Bertani (LB) broth or on LB agar and used for routine DNA manipulation.

*Rhodotorula toruloides* was cultured in lipid production medium MinRL3 unless indicated otherwise. Medium MinRL3 contains (per l) 70 g glucose, 1.5 g yeast extract, 0.5 g (NH_4_)_2_SO_4_, 2.05 g K_2_HPO_4_, 1.45 g KH_2_PO_4_, 0.6 g MgSO_4_, 0.3 g NaCl, 10 mg CaCl_2_, 1 mg FeSO_4_, 0.5 mg ZnSO_4_, 0.5 mg CuSO_4_, 0.5 mg H_3_BO_4_, 0.5 mg MnSO_4_, 0.5 mg NaMoO_4_, with pH adjusted to 6.1.

In some experiments, GJ2013 medium [39] was used as another lipid production medium. Medium GJ2013 (per l) contains 40 g glucose, 0.4 g KH_2_PO_4_, 1.5 g MgSO_4_·7H_2_O, 10 mL TE solution, pH6.0. TE solution (per litre) contains 4.0 g CaCl_2_·2H_2_O, 0.55 g FeSO_4_·7H_2_O, 0.52 g citric acid·H_2_O, 0.1 g ZnSO_4_·7H_2_O, 0.076 g MnSO_4_·H_2_O, 0.1 mL smoked H_2_SO_4_ [40].

### Plasmid construction

Oligonucleotides used are listed in Table 2. All DNA restriction and modification enzymes were sourced from New England Biolabs (NEB, MA, USA). Plasmid pKCL2 (Fig. 5a) is similar to pKC2 except that the Rt*GFP* fragment was replaced with Rt*LUC2* gene [12]. This allowed efficient intergation of reporter gene cassette at the CAR2 locus, consisting of a hygromycin resistant cassette (P_*GPD1*–*3*_::*HPT*-*3*::T_*SV40*_) and a luciferase reporter cassette (P_*GPD1*_::Rt*LUC2*::T_*35S*_) flanked by *CAR2* sequence [8]. P_*GPD1*–*3*_ and P_*GPD1*_ are the glyceraldehyde 3-phosphate dehydrogenase promoter of *R. graminis* WP1 and *R. toruloides* ATCC 10657, with GenBank accession number of JQ806386 and JN208861, respectively [7]. *HPT*-*3* (JQ806387) and Rt*LUC2* (KR258785) are codon-optimized synthetic genes encoding *E. coli* hygromycin phosphotransferase and firefly luciferase (Luc2, ACH53166), respectively [7].

**Table 2:** Sequences of oligonucleotides

Name	Sequence (5′-3′)	Information
SV40R	TTT*ccgcgg*TCGAATTTCCCCGATCGTTCA	*T*_*SV40*_
LUC2U	GAGTCGCTCACCTACTGCATC	*RtLUC2*
ACC1U1	GAAGGCGGGGGTTCTCGGAAG	*ACC1*
LDP1U1	GACGAGGTCATCCGCGAG	*LDP1 5′UTR*
LDP1L1	GACCAGCTCTACCAGCGCATCAC	*LDP1 3′UTR*
CRP79L1	TCGCCCTCCTCCCCTGCTCGCAAAT	*CRP79*
Rt232Sf	TTT*actagt*GGTCGCTTCTTTCCTCGCAG	*ACC1/ACC1in*
Rt233Nr	TTT*ccatgg*GAAGTGAAGTTGGGGAACG	*ACC1*
Rt310Nr	TTT*ccatgg*AGAACCTGTCGTCGCATGA	*ACC1in*
Rt359Sf	TTT*actagt*TCGACTTGTCTTCCTCCGCGA	*DUR1/DRU1in*
Rt360Nr	TTT*ccatgg*CGAAAGAGGGATGTGAG	*DUR1*
Rt424Nr	TTT*ccatgg*AGAAGAGGTTCTGCGCGGA	*DUR1in*
Rt363Sf	TTT*actagt*CTGTGATGCTAGGTGTCGATC	*ACL1/ACL1in*
Rt364Nr	TTT*ccatgg*CTGCTGCGTTTCCTGGTAC	*ACL1*
Rt365Nr	TTT*ccatgg*CGTCGTACTCGCGGATG	*ACL1in*
Rt369Sf	TTT*actagt*GAACTCGACTCATTACGGGAG	*FAS1/FAS1in*
Rt370Nr	TTT*ccatgg*TGTGCGGTATTCGACGAGTTTG	*FAS1*
Rt371Nr -1	TTT*ccatgg*AGTAGTGCTGTCCGCGCAGA	*FAS1in*
Rt361Sf	TTT*actagt*CTCTAGCCTACGACCGCCTC	*FAT1/FAT1in*
Rt362Nr	TTT*ccatgg*TAGCGAGTCGTTCTCTGCAG	*FAT1*
Rt516Nr	TTT*ccatgg*GCGAGGCGGTTGACCTCTGC	*FAT1in*
Rt359Sf	TTT*actagt*TCGACTTGTCTTCCTCCGCGA	*DUR1/DUR1in*
Rt360Nr	TTT*ccatgg*CGAAAGAGGGATGTGAG	*DUR1*
Rt424Nr	TTT*ccatgg*AGAAGAGGTTCTGCGCGGA	*DUR1in*
Rt366Sf	TTT*actagt*CACGCCTCTGTGACTCGGTAC	*LDP1/LDP1in*
Rt367Nr	TTT*ccatgg*CGTGCGAGTGTGCGTGCGA	*LDP1*
Rt368Nr	TTT*ccatgg*GGTAGTCCGACACCTGCG	*LDP1in*

All promoter sequences were amplified using *R. toruloides* genomic DNA as the template unless indicated otherwise. Upstream sequence of *ACL1* (−1000 to −1) and *ACL1in* (−1000 to +167) was amplified using oligo pair Rt363Sf/Rt364Nr and Rt363Sf/Rt365Nr, respectively. The resulting PCR products of *ACL1* and *ACL1in* promoter were double digested with SpeI and NcoI and inserted to the same sites of vector pKCL2 to create plasmid pKCLAL1 and pKCLAL2, respectively. Similarly, upstream sequence of *FAS1* (−1001 to −1), *FAS1in* (−1001 to +271), *FAT1* (−1003 to −1), *FAT1in* (−1003 to +417), *DUR1* (−493 to −1), *DUR1in* (−493 to +627), *ACC1* (−1501 to −1), *ACC1in* (−1501 to +91), *LDP1* (−362 to −1) and *LDP1in* (−362 to +152) was amplified using oligo pair Rt369Sf/Rt370Nr, Rt369Sf/Rt371Nr, Rt361Sf/Rt362Nr, Rt361Sf/Rt516Nr, Rt359Sf/Rt360Nr, Rt359Sf/Rt424Nr, Rt232Sf/Rt233Nr, Rt232Sf/Rt310Nr, Rt366Sf/Rt367Nr and Rt366Sf/Rt368Nr (Table 2) to create pKCLF3, pKCLF4, pKCLF5, pKCLF6, pKCLDU1, pKCLDU2, pKCLA1, pKCLA2, pKCLP3 and pKCLP4, respectively.

### *Agrobacterium tumefaciens*-mediated transformation

The binary vectors were electroporated into *A. tumefaciens* AGL1 (2.5 kV, 25 μF, 400 Ω) and subsequently selected with 2YT agar medium supplemented with streptomycin (100 µg/ml). Fungi transformation via ATMT was performed as described previously [7].

### Isolation of genomic DNA and total RNA

Genomic DNA and RNA of *R. toruloides* were extracted as described previously [7]. The concentration and purity of the extracted DNA and RNA were analyzed using NanoDrop® ND-1000 Spectrophotometer (Nanodrop Technologies, USA) and agarose gel electrophoresis.

### Gene annotation in *R. toruloides*

Due to the very high sequence homology between *R. toruloides* ATCC 10657 and *R. glutinis* ATCC 204091, genome database of *R. glutinis* ATCC 204091 was used as the reference unless indicated otherwise. The putative encoding gene of acetyl-CoA carboxylase (*ACC1*), ATP:citrate lyase (*ACL1*), β subunit of fatty acid synthetase (*FAS1*), fatty acid transporter (*FAT1*), urea amidolyase (*DUR1*) and perilipin (*LDP1*) were annotated using orthologous sequences from *S. cerevisiae*, *Y. lipolytica* or *R. toruloides* NP11 (Table 1).

### Rapid amplification of cDNA ends (RACE)

The 5′ and 3′ end of cDNA sequences were determined by 5′ RACE and 3′ RACE using BD SMARTer™ RACE cDNA Amplification Kit (Clontech, CA, USA) according to the manufacturer’s instruction. Oligos ACC1U1 (Table 2) was used as the specific primer for 5′ RACE of *ACC1*, LDP1U1 and LDP1L1 (Table 2) was used for 5′ RACE and 3′ RACE of *LDP1*, respectively.

### Quantitative reverse transcription PCR (qRT-PCR)

Total RNA was treated with DNase I (Roche Diagnostics, Mannheim, Germany) followed by precipitation with ethanol to remove trace amount of DNA. cDNA was synthesized by reverse transcription using iScript™ Reverse Transcription Supermix (Bio-Rad Laboratories, CA, USA). Real-time PCR was conducted in ABI PRISM 7900HT Sequence Detection System (Applied Biosystems, CA, USA) using the ABI SYBR® Select Master Mix (Applied Biosystems). PCR conditions were as follows: an initial 50 °C for 2 min and 95 °C denaturation step for 10 min followed by 40 cycles of denaturation at 95 °C for 15 s, annealing at 60 °C for 1 min. Triplicates were used for all analysis. The data was acquired using the software SDS 2.4 (Applied Biosystems) and relative gene expression levels were calculated against the reference encoding gene of actin (*ACT1*, GenBank acc. no. KR138696) [12] using the 2^−ΔΔCt^ method through the RQ Manager software v1.2.1 (Applied Biosystems).

### Reporter assay

Strains bearing the integrated T-DNA at the CAR2 locus were identified by the albino phenotype followed by Southern blot verification. The strains were then cultured in YPD broth to mid-exponential phase and used for luciferase assay. Cells were washed twice with water and inoculated to the indicated medium at an optical density (OD_600_) of 0.5 and cultured at 30 °C with agitation (250 rpm). Luciferase activity was determined by one-step measurement method as described previously [7] with some modifications. Briefly, cell culture (10 µl) was mixed with 85 µl of PBS buffer (pH7.4) and 5 µl of 10 mM D-luciferin (DMSO solution, catalogue No. L9504, Sigma-Aldrich), loaded in a well of FluoroNunc 96-well plate (Thermo Fisher Scientific, Langenselbold, Germany) for measurement of bioluminescence. Cell density was measured at OD of 600 nm with 10-20 fold dilution of cell culture in PBS buffer to a final volume of 100 μl, and loaded to a well of 96-well flat-bottom transparent plate (Nunc, Roskilde, Denmark). All data was measured and acquired with the Tecan Infinite M200 plate reader coupled with the iCycler version 3.0 software (Tecan, Salzburg, Austria). The relative promoter activity (RPA) was calculated by normalization against that of *GPD1* promoter.

## Additional files

**Additional file 1.** Promoter sequences. Promoter range in the gene was shown in the parenthesis and substituted sequences are in red font. Translational starts (ATG) are underlined.

**Additional file 2.** Comparison of intronic and intronless *GPD1* promoter strength. Luciferase gene assay was performed using the reporter strain with 795 and 932 bp promoter of *GPD1* (−795 to +1) and *GPD1in* (−795 to +137), respectively. Cells were cultured in MinRL3 medium for 4 days and luciferase reporter assay was performed daily.

## Abbreviations

UTR: untranslated region
Are1: acyl-CoA:sterol acyltransferase
ATCC: American type culture collection, USA
BLAST: basic local alignment search tool (National Library of Medicine, National Institutes of Health, USA)
*CAR2*: bifunctional enzyme phytoene synthase and lycopene cyclase encoding gene
Dga1: acyl-CoA:diacylglycerol acyltransferase
Dga3: soluble diacylglycerol acyltransferase
*GPD1*: glyceraldehyde 3-phosphate dehydrogenase gene
IME: intron-mediated enhancement
Lro1: phospholipid:diacylglycerol acyltransferase
qPCR: quantitative reverse transcription polymerase chain reaction
RACE: rapid amplification of cDNA ends
Rt*LUC2*: a commercially synthesized firefly luciferase protein (Luc2) gene according to the codon bias of *R. toruloides*
TAG: triacylglycerol
TSS: transcriptional start site

## References

[1] Wang QM, Yurkov AM, Goker M, Lumbsch HT, Leavitt SD, Groenewald M, Theelen B, Liu XZ, Boekhout T, Bai FY (2015). Phylogenetic classification of yeasts and related taxa within *Pucciniomycotina*. Stud Mycol.

[2] Sampaio JP, Gadanho M, Bauer R, Weiß M (2003). Taxonomic studies in the Microbotryomycetidae: *Leucosporidium golubevii* sp. nov., *Leucosporidiella* gen. nov. and the new orders Leucosporidiales and Sporidiobolales. Mycol Prog.

[3] Yamazaki M, Komagata K (1981). Taxonomic significance of electrophoretic comparison of enzymes in the genera *Rhodotorula* and *Rhodosporidium*. Int J Syst Bacteriol.

[4] Zhao X, Wu S, Hu C, Wang Q, Hua Y, Zhao ZK (2010). Lipid production from Jerusalem artichoke by *Rhodosporidium toruloides* Y4. J Ind Microbiol Biotechnol.

[5] Liu H, Zhao X, Wang F, Li Y, Jiang X, Ye M, Zhao ZK, Zou H (2009). Comparative proteomic analysis of *Rhodosporidium toruloides* during lipid accumulation. Yeast.

[6] Turcotte G, Kosaric N (1988). Biosynthesis of lipids by *Rhodosporidium toruloides* ATCC 10788. J Biotechnol.

[7] Liu Y, Koh CM, Sun L, Hlaing MM, Du M, Peng N, Ji L (2013). Characterization of glyceraldehyde-3-phosphate dehydrogenase gene Rt*GPD1* and development of genetic transformation method by dominant selection in oleaginous yeast *Rhodosporidium toruloides*. Appl Microbiol Biotechnol.

[8] Koh CM, Liu Y, Du Moehninsi M, Ji L (2014). Molecular characterization of *KU70* and *KU80* homologues and exploitation of a *KU70*-deficient mutant for improving gene deletion frequency in *Rhodosporidium toruloides*. BMC Microbiol.

[9] Lin X, Wang Y, Zhang S, Zhu Z, Zhou YJ, Yang F, Sun W, Wang X, Zhao ZK (2014). Functional integration of multiple genes into the genome of the oleaginous yeast *Rhodosporidium toruloides*. FEMS Yeast Res.

[10] Abbott EP, Ianiri G, Castoria R, Idnurm A (2013). Overcoming recalcitrant transformation and gene manipulation in *Pucciniomycotina* yeasts. Appl Microbiol Biotechnol.

[11] Wang Y, Lin X, Zhang S, Sun W, Ma S, Zhao ZK (2016). Cloning and evaluation of different constitutive promoters in the oleaginous yeast *Rhodosporidium toruloides*. Yeast.

[12] Liu Y, Koh CMJ, Ngoh ST, Ji L (2015). Engineering an efficient and tight D-amino acid-inducible gene expression system in *Rhodosporidium/Rhodotorula* species. Microb Cell Fact.

[13] Tai M, Stephanopoulos G (2013). Engineering the push and pull of lipid biosynthesis in oleaginous yeast *Yarrowia lipolytica* for biofuel production. Metab Eng.

[14] Zhu Z, Zhang S, Liu H, Shen H, Lin X, Yang F, Zhou YJ, Jin G, Ye M, Zou H, Zhao ZK (2012). A multi-omic map of the lipid-producing yeast *Rhodosporidium toruloides*. Nat Commun.

[15] Zhu Z, Ding Y, Gong Z, Yang L, Zhang S, Zhang C, Lin X, Shen H, Zou H, Xie Z (2015). Dynamics of the Lipid Droplet Proteome of the Oleaginous Yeast *Rhodosporidium toruloides*. Eukaryot Cell.

[16] Digel M, Ehehalt R, Fullekrug J (2010). Lipid droplets lighting up: insights from live microscopy. FEBS Lett.

[17] Kimmel AR, Brasaemle DL, McAndrews-Hill M, Sztalryd C, Londos C (2010). Adoption of PERILIPIN as a unifying nomenclature for the mammalian PAT-family of intracellular lipid storage droplet proteins. J Lipid Res.

[18] Athenstaedt K, Jolivet P, Boulard C, Zivy M, Negroni L, Nicaud JM, Chardot T (2006). Lipid particle composition of the yeast *Yarrowia lipolytica* depends on the carbon source. Proteomics.

[19] Wang C, St Leger RJ (2007). The *Metarhizium anisopliae* Perilipin Homolog MPL1 Regulates Lipid Metabolism, Appressorial Turgor Pressure, and Virulence. J Biol Chem.

[20] Roy SW (2006). Intron-rich ancestors. Trends Genet.

[21] Rose AB (2008). Intron-mediated regulation of gene expression. Curr Top Microbiol Immunol.

[22] Mascarenhas D, Mettler IJ, Pierce DA, Lowe HW (1990). Intron-mediated enhancement of heterologous gene expression in maize. Plant Mol Biol.

[23] Duncker BP, Davies PL, Walker VK (1997). Introns boost transgene expression in *Drosophila melanogaster*. Mol Gen Genet.

[24] Lugones LG, Scholtmeijer K, Klootwijk R, Wessels JG (1999). Introns are necessary for mRNA accumulation in *Schizophyllum commune*. Mol Microbiol.

[25] Xu J, Gong ZZ (2003). Intron requirement for AFP gene expression in *Trichoderma viride*. Microbiology.

[26] Hong SP, Seip J, Walters-Pollak D, Rupert R, Jackson R, Xue Z, Zhu Q (2012). Engineering *Yarrowia lipolytica* to express secretory invertase with strong *FBA1IN* promoter. Yeast.

[27] Hu J, Ji L (2016). Draft genome sequences of *Rhodosporidium toruloides* strains ATCC 10788 and ATCC 10657 with compatible mating types. Genome Announc.

[28] Paul D, Magbanua Z, Arick M, French T, Bridges SM, Burgess SC, Lawrence ML (2014). Genome Sequence of the Oleaginous Yeast *Rhodotorula glutinis* ATCC 204091. Genome Announc.

[29] Gagniuc P, Ionescu-Tirgoviste C (2012). Eukaryotic genomes may exhibit up to 10 generic classes of gene promoters. BMC Genom.

[30] Leavitt JM, Alper HS (2015). Advances and current limitations in transcript-level control of gene expression. Curr Opin Biotechnol.

[31] Juneau K, Miranda M, Hillenmeyer ME, Nislow C, Davis RW (2006). Introns regulate RNA and protein abundance in yeast. Genetics.

[32] Bornstein P, McKay J (1988). The first intron of the α1 (I) collagen gene contains several transcriptional regulatory elements. J Biol Chem.

[33] Aronow B, Lattier D, Silbiger R, Dusing M, Hutton J, Jones G, Stock J, McNeish J, Potter S, Witte D, Wiginton D (1989). Evidence for a complex regulatory array in the first intron of the human adenosine deaminase gene. Genes Dev.

[34] Rippe RA, Lorenzen SI, Brenner DA, Breindl M (1989). Regulatory elements in the 5′-flanking region and the first intron contribute to transcriptional control of the mouse alpha 1 type I collagen gene. Mol Cell Biol.

[35] Son GH, Jung H, Seong JY, Choe Y, Geum D, Kim K (2003). Excision of the first intron from the gonadotropin-releasing hormone (GnRH) transcript serves as a key regulatory step for GnRH biosynthesis. J Biol Chem.

[36] Bossu JP, Chartier FL, Fruchart JC, Auwerx J, Staels B, Laine B (1996). Two regulatory elements of similar structure and placed in tandem account for the repressive activity of the first intron of the human apolipoprotein A-II gene. Biochem J.

[37] Gallegos JE, Rose AB (2015). The enduring mystery of intron-mediated enhancement. Plant Sci.

[38] Lazo GR, Stein PA, Ludwig RA (1991). A DNA transformation-competent *Arabidopsis* genomic library in *Agrobacterium*. Biotechnology (N Y).

[39] Jin G, Zhang Y, Shen H, Yang X, Xie H, Zhao ZK (2013). Fatty acid ethyl esters production in aqueous phase by the oleaginous yeast *Rhodosporidium toruloides*. Bioresour Technol.

[40] Meesters PA, Eggink G (1996). Isolation and characterization of a delta-9 fatty acid desaturase gene from the oleaginous yeast *Cryptococcus curvatus* CBS 570. Yeast.

