# Supplementary material for: Developing a set of strong intronic promoters for robust metabolic engineering in oleaginous Rhodotorula (Rhodosporidium) yeast species
Source: Microb Cell Fact. 2016 Nov 25;15:200. doi: 10.1186/s12934-016-0600-x (PMC5124236; doi:10.1186/s12934-016-0600-x)
Supplement: Supplementary file 1 — Additional file 1. Promoter sequences. Promoter range in the gene was shown in the parenthesis and substituted sequences are in red font. Translational starts (ATG) are underlined. [file 12934_2016_600_MOESM1_ESM.docx]

**Additional file 1. Promoter sequences.** Promoter range in the gene was shown in the parenthesis and mutation sequences are in **red** font. Translation start codons are underlined.

>P*_ACC1_* (-1501 ~ +3 of *ACC1* gene)

ggtcgcttctttcctcgcagcacgcttttgtcggctccctgatcagcacacaagctaactaacgctctggtttcgctggcagtcatgcacggccttggctcgtcaacctcgttctgggaagcgcccttctcccgctcaaacctgtcctcccgcttccgcctcatccgctacgacttcgacggccacggtctctcgcccgtctcgtccctcgacgcagcagatgacggcgccatgatcccgctcgacgacctcgtcggggacttggcggctgtgatcgagtgggctggggtggagaaggttgcgggagttgttggacactcgatgagcgggctggtggcgagcacatttgcggccaagtacccgcagaagctcgacaagctcggtgagtcgcattgaaccttcctccgccgtctcttctccgctgacgattcgtcgacttggccctgcttctcgcgcagtcctcctcggcgcaatgcgctctctgaaccctaccgtccaaagcaacatgctcaagcgagccgatacagtcctcgaatccggcctctcagcaatcgtcgcacaagtcgtctccgccgctttgtccgacaagtcaaagcaggactcgcccctctcggcagcgatggtgcgaacgctcgtgcttggaacggacccgagagggtacgcggcggcgtgtagggcgcttgcgggtgcgaaggacccggattactcgagcatcaaggccgagacgttgggtgcgttcgcttgttctccttcctctgcttttctcccagcaactgacgcaagcgtctgcaacacagtcgtcgcaggcgagtttgactacctctcgaacaaggagacgaccgacgcgctggtcaacgacatcccgggcgcggagaaggtccagatggacagtgtcggccactggcacgccgtcgaggaccccgttggactcgccaagatcctcgatgggttcttcttgcaggggaaatgaggttgggaaggggggatagactggggagaacggcaggtgcgtacgcagcggacgtcggtcgggaggactttttcggggaggatattcgctgactgactccgacgtcgctttcctccttgcagtatcttcagaagggatgggaggaggcgaactgcaagggtaatgaacgagacaacgccgagggaggaagcgccggaactctcgggggcgaagaaggagtggtgtcttcgccagcgaacagcttccggggtgggttggacagcgccagtagaattccagcgtcgcaacagagctctagtcgaccgcgatcacccacaaggacgagagcgggtcgcgccttgtccgcttccccatcctcgtcctgctcttgctctcttccctaccacactctcccgcttgcgggctctctttctcgcttggcgctcctgctaccgctactctagactctcctagtctccctgcacaaccatccctatcccctccgcctctctcgcacaccccccacagcttcgttccccaacttcacttc**c**atg

>P*_ACC1in_* (-1501 ~ +95 of *ACC1* gene)

ggtcgcttctttcctcgcagcacgcttttgtcggctccctgatcagcacacaagctaactaacgctctggtttcgctggcagtcatgcacggccttggctcgtcaacctcgttctgggaagcgcccttctcccgctcaaacctgtcctcccgcttccgcctcatccgctacgacttcgacggccacggtctctcgcccgtctcgtccctcgacgcagcagatgacggcgccatgatcccgctcgacgacctcgtcggggacttggcggctgtgatcgagtgggctggggtggagaaggttgcgggagttgttggacactcgatgagcgggctggtggcgagcacatttgcggccaagtacccgcagaagctcgacaagctcggtgagtcgcattgaaccttcctccgccgtctcttctccgctgacgattcgtcgacttggccctgcttctcgcgcagtcctcctcggcgcaatgcgctctctgaaccctaccgtccaaagcaacatgctcaagcgagccgatacagtcctcgaatccggcctctcagcaatcgtcgcacaagtcgtctccgccgctttgtccgacaagtcaaagcaggactcgcccctctcggcagcgatggtgcgaacgctcgtgcttggaacggacccgagagggtacgcggcggcgtgtagggcgcttgcgggtgcgaaggacccggattactcgagcatcaaggccgagacgttgggtgcgttcgcttgttctccttcctctgcttttctcccagcaactgacgcaagcgtctgcaacacagtcgtcgcaggcgagtttgactacctctcgaacaaggagacgaccgacgcgctggtcaacgacatcccgggcgcggagaaggtccagatggacagtgtcggccactggcacgccgtcgaggaccccgttggactcgccaagatcctcgatgggttcttcttgcaggggaaatgaggttgggaaggggggatagactggggagaacggcaggtgcgtacgcagcggacgtcggtcgggaggactttttcggggaggatattcgctgactgactccgacgtcgctttcctccttgcagtatcttcagaagggatgggaggaggcgaactgcaagggtaatgaacgagacaacgccgagggaggaagcgccggaactctcgggggcgaagaaggagtggtgtcttcgccagcgaacagcttccggggtgggttggacagcgccagtagaattccagcgtcgcaacagagctctagtcgaccgcgatcacccacaaggacgagagcgggtcgcgccttgtccgcttccccatcctcgtcctgctcttgctctcttccctaccacactctcccgcttgcgggctctctttctcgcttggcgctcctgctaccgctactctagactctcctagtctccctgcacaaccatccctatcccctccgcctctctcgcacaccccccacagcttcgttccccaacttcacttccgatgccgtgcgtcgcctccctttcgcctggcgggcccgcgcctgcttccgaggacaactactgattgtgggatcatgcgacgacaggttctc**catg**

>P*_ACC1inm_* (-201 ~ +95 of *ACC1* gene)

gtcgcgccttgtccgcttccccatcctcgtcctgctcttgctctcttccctaccacactctcccgcttgcgggctctctttctcgcttggcgctcctgctaccgctactctagactctcctagtctccctgcacaaccatccctatcccctccgcctctctcgcacaccccccacagcttcgttccccaacttcacttccgat**c**ccgtgcgtcgcctccctttcgcctggcgggcccgcgcctgcttccgaggacaactactgattgtgggatcatgcgacgacaggttctc**catg**

>P*_ACL1_* (-1000 ~ +3 of *ACL1* gene)

ctgtgatgctaggtgtcgatcgagggaggaggtggacgaggagaagccagtctgagcgaagttgacatccgcctcatctctccctctcgctgcgctcgtctcgctcttcacgtcctcagcctcacaacgtcgagtaccaccagcagcaagctcagcaactctgcacggcgccgcctgttcgcccgagtgttcggagcggtgggaggaactcctcggcctttcgcagctggtcgtccacgagtcgctggaggatgaaactttcggtcgtggtcaacagcgtccttacaacgtcgcgtggcaagtatcaagcgaaaagagcgtggtgacgaggggtgagagcggttgaaagaagcggagggtcggagcgattcgcggtgttgcaacggcggcggggaaagttgcttgcgctccgtcgtctggctctcttgcttcctactactcgctagtacccagtacaagctactacaggctatgcagactcctactcgcctgctacagcttgcgcactatgacttcgtccccctcctccgctcgaccactcgtcggactcgcctcacacgacggcgacaacgcctggccgccctcgctccgactgggtacagcagaggaggacccgctggtggactttcgggggtcgagacaccgcggcactgtacttcactacgcccatcctctcgacggtggctgtatgcggtgatgtcccctcgctcctgggccgcctgctgtccctcggccgcaggacgcgtccttgcgcccgttggagcgtgtaacttgctcgaatacgcgcatctagcacacacgcactgctactgagcttgcacgaggcgacctgctcgctcgggcccccaacagcccttcacccgcctcgttcgacctcgactttcctttcttccttccactcctcactccgctcacctcgagcaacctcaaacagactcgcttcggcttctccttccttctataccccccaaccaccgacgtaccaggaaacgcagcagc**c**atg

>P*_ACL1in_* (-1000 ~ +167 of *ACL1* gene)

ctgtgatgctaggtgtcgatcgagggaggaggtggacgaggagaagccagtctgagcgaagttgacatccgcctcatctctccctctcgctgcgctcgtctcgctcttcacgtcctcagcctcacaacgtcgagtaccaccagcagcaagctcagcaactctgcacggcgccgcctgttcgcccgagtgttcggagcggtgggaggaactcctcggcctttcgcagctggtcgtccacgagtcgctggaggatgaaactttcggtcgtggtcaacagcgtccttacaacgtcgcgtggcaagtatcaagcgaaaagagcgtggtgacgaggggtgagagcggttgaaagaagcggagggtcggagcgattcgcggtgttgcaacggcggcggggaaagttgcttgcgctccgtcgtctggctctcttgcttcctactactcgctagtacccagtacaagctactacaggctatgcagactcctactcgcctgctacagcttgcgcactatgacttcgtccccctcctccgctcgaccactcgtcggactcgcctcacacgacggcgacaacgcctggccgccctcgctccgactgggtacagcagaggaggacccgctggtggactttcgggggtcgagacaccgcggcactgtacttcactacgcccatcctctcgacggtggctgtatgcggtgatgtcccctcgctcctgggccgcctgctgtccctcggccgcaggacgcgtccttgcgcccgttggagcgtgtaacttgctcgaatacgcgcatctagcacacacgcactgctactgagcttgcacgaggcgacctgctcgctcgggcccccaacagcccttcacccgcctcgttcgacctcgactttcctttcttccttccactcctcactccgctcacctcgagcaacctcaaacagactcgcttcggcttctccttccttctataccccccaaccaccgacgtaccaggaaacgcagcagcaatgtcggcgaaggtgcgtcgaatcgccttcccgcgacctcgagacgacattcctgactttctccctccctatccctcttctgtcctctctgcctaatccttgatgcctctaccatcccactcgacgactcacgaacccacccgcagcccatccgcgagtacgacgccatg

>P*_FAS1_* (-739 ~ +3 of *FAS1* gene)

gaactcgactcattacgggaggcgccgacaagacgatcaaggttcgtccaggacagcccttctctgccgtcgagtttcgttcccgctgacaccctgccttgtgcgcagatctactctgagcaagcataggtcgttccagctgtaccggcgcggtgatcgtcgttgtgcgagtgtaacattgtgcgatacccagcagcctatcggaggacagcgagtgcctcgagagctggagacgaggagtcgggtgaagcgggcaaggctggcctcgctggatgctgagacgtcccacagcatcgtgcacgaaggaggaggggacgggcggggacgaagcggtgttggtcgtcgggacctcgtcgtccgaaagttgggtcgtcgctccgtcgcgcggctcgcttctctcgtttccttctcttccacccgctcgctctgcttcttgcttgaactggctcagcttggctcgctaggaacgaactactcgctacactaccccaggtgcgcaacggacttcccactctccagctctcggacctcgacgagcgaaagacgaaccccaacgacctctcctctcgcctgcctcgtcttgctcagcacctaacgacacacgagcgacccagcccgactagactcgcgcaaacctcgcaaactgacccgcttgccattcgcctctccagctccctctccccgtcctcgtctccccacttcgctaccctctcttcaaactcgtcgaataccgcaca**cc**atg

>P*_FAS1in_* (-739 ~ +271 of *FAS1* gene)

gaactcgactcattacgggaggcgccgacaagacgatcaaggttcgtccaggacagcccttctctgccgtcgagtttcgttcccgctgacaccctgccttgtgcgcagatctactctgagcaagcataggtcgttccagctgtaccggcgcggtgatcgtcgttgtgcgagtgtaacattgtgcgatacccagcagcctatcggaggacagcgagtgcctcgagagctggagacgaggagtcgggtgaagcgggcaaggctggcctcgctggatgctgagacgtcccacagcatcgtgcacgaaggaggaggggacgggcggggacgaagcggtgttggtcgtcgggacctcgtcgtccgaaagttgggtcgtcgctccgtcgcgcggctcgcttctctcgtttccttctcttccacccgctcgctctgcttcttgcttgaactggctcagcttggctcgctaggaacgaactactcgctacactaccccaggtgcgcaacggacttcccactctccagctctcggacctcgacgagcgaaagacgaaccccaacgacctctcctctcgcctgcctcgtcttgctcagcacctaacgacacacgagcgacccagcccgactagactcgcgcaaacctcgcaaactgacccgcttgccattcgcctctccagctccctctccccgtcctcgtctccccacttcgctaccctctcttcaaactcgtcgaataccgcacaggatggttgcggcgcaggagttgccgcttgcgctgagcatcagcttcgcgcccgagtcgtcgaccatctcgatgacgctgttcaaccagcccgagacgtcgaaacccgccctccccctcgagctcaagtacaagtacgacccctcgacgccgtacgccccgatccacgagatcaccgaggaccgtaaccagaggatcaagcaggtgcgcgaaaggccgttcaagcgaagggcgagcgagaactgatgaatttctgcgcggacagcactact**cc**atg

>P*_FAT1_* (-1003~+3 of *FAT1* gene)

ctctagcctacgaccgcctcatcctgcgccgcctgtcctgcagtggcgtattgcttttcgcaccagctacgatctccgctggacttcccgggctactcctcctgggaatgctgcgagctgttgggtgaccgagtcgggcgcaaaggaggtcggggggagaatgggcggcccgtctcgctctctcacgcctcagaacgcccagctgaagctttgcccggacgtatgactacttcagcgagtagcccagcttttcagcaggcaaaggatgtgtgtagtgaggatcggggtgtcgacctccccttctcctccccctcgtcacctctctcgctcgtcacaccaccagaatgggtctttcaatctctcgcctgtgtcagttggttctctgagctctgggcgccttgttccagcccacctggaggcttgggggacgagattggcgccgtcaaagccaggagtgttggcgcaccgtcccccgtctcgcgcacgcctctgggacggccgctcaggatctcgtactgactactgaactcctgcatgtacgcctgaaccatccacgcctctccccgccaccgccatcgcttgctgttgaccgtctccccctccaacacgcgtcgctacgtctggctcgacttacctgattgcgctcactcgtcgcgcgaatggacgactgtgcactcgcttgattgcctacacccgttgtggctgtcgacattccaggtgaaggggaggaggaaggggaaggttcgaagaagaccttgcaatggacggcggtctgctccgtccacagggcgttgagtctccgtcgttccggagcacaagcacggcgtgcgcacgttctcagcgagaaggacagcaagcgtttgcgagtgaaagggggacgttcgagttggccggcctgagggacttcgccgagagcgacaccgccttcgccgtctcacgctcgtcgctctctcgctctcgcgctttctgaggcttgtactgctgcagagaacgactcgctac**c**atg

>P*_FAT1in_* (-1003~+417 of *FAT1* gene)

ctctagcctacgaccgcctcatcctgcgccgcctgtcctgcagtggcgtattgcttttcgcaccagctacgatctccgctggacttcccgggctactcctcctgggaatgctgcgagctgttgggtgaccgagtcgggcgcaaaggaggtcggggggagaatgggcggcccgtctcgctctctcacgcctcagaacgcccagctgaagctttgcccggacgtatgactacttcagcgagtagcccagcttttcagcaggcaaaggatgtgtgtagtgaggatcggggtgtcgacctccccttctcctccccctcgtcacctctctcgctcgtcacaccaccagaatgggtctttcaatctctcgcctgtgtcagttggttctctgagctctgggcgccttgttccagcccacctggaggcttgggggacgagattggcgccgtcaaagccaggagtgttggcgcaccgtcccccgtctcgcgcacgcctctgggacggccgctcaggatctcgtactgactactgaactcctgcatgtacgcctgaaccatccacgcctctccccgccaccgccatcgcttgctgttgaccgtctccccctccaacacgcgtcgctacgtctggctcgacttacctgattgcgctcactcgtcgcgcgaatggacgactgtgcactcgcttgattgcctacacccgttgtggctgtcgacattccaggtgaaggggaggaggaaggggaaggttcgaagaagaccttgcaatggacggcggtctgctccgtccacagggcgttgagtctccgtcgttccggagcacaagcacggcgtgcgcacgttctcagcgagaaggacagcaagcgtttgcgagtgaaagggggacgttcgagttggccggcctgagggacttcgccgagagcgacaccgccttcgccgtctcacgctcgtcgctctctcgctctcgcgctttctgaggcttgtactgctgcagagaacgactcgctacgatggcactcccagcgctcggaccggccgcaacagcgctcgcaggcgccagctcgttcatgtacctcgacgccctctggcgcatgggcgaggactacaagctcgctcgcggaatcgtcaaggcccgcatcgccctcgctctcaacaacaggagggacaggaactcgatctactatgtgtttgacgacgcgcacaggaagaggggagatgccgactgttacgtctgcgacggggtcacatacagctggaaccaggtcgcgctcggtgagttggcattcgtcagtactctcaaacaaggcttcgccatcccctgctactgccacaccctcccccgagctgacctcgtcgccttcctcctcctctccccacttcaccggtccactcgaaatcactgctcgcagaggtcaaccgcctcgc**c**c**atg**

>P*_DUR1_* (-493~+3 of *DUR1* gene)

ttcgacttgtcttcctccgcgactcgttctgttgctccgcaactcccgctcttagccgcgccgctatctctgcgatactcagacgaaacgcttgagctttctgccagtcgacccgtgacgattccgcccccgcttggcggctacgcctcttccccgcttctcctcgtcctttccgagggttgttgctgccgtcaacgtccagcctgcgcttcactggactgccgttggagctcaaacgctactggccctctagtcaactcgcctagcgactccagcgcagccctcgctctgcttcagacccttccccacttcctcccctttcttcgatctcgcaaacagcgacaacaccttccgccagccgcacgacaagcttgacttcaccctaaacctctcatcttatcagcacaagacccccgcgaaactcgcatcagcgaccactcactccaaccgacgaacccctccctccgaccctccctcacatccctctttcgccatg

>P*_DUR1in_* (-493~+630 of *DUR1* gene)

ttcgacttgtcttcctccgcgactcgttctgttgctccgcaactcccgctcttagccgcgccgctatctctgcgatactcagacgaaacgcttgagctttctgccagtcgacccgtgacgattccgcccccgcttggcggctacgcctcttccccgcttctcctcgtcctttccgagggttgttgctgccgtcaacgtccagcctgcgcttcactggactgccgttggagctcaaacgctactggccctctagtcaactcgcctagcgactccagcgcagccctcgctctgcttcagacccttccccacttcctcccctttcttcgatctcgcaaacagcgacaacaccttccgccagccgcacgacaagcttgacttcaccctaaacctctcatcttatcagcacaagacccccgcgaaactcgcatcagcgaccactcactccaaccgacgaacccctccctccgaccctccctcacatccctctttcgccatgtccgcgacaaagcagcacaagctcctcatcgccaaccgcggcgagatcgctttgcgatgcatgcgttcagctgccgcactcaagatcccgaccgtcgccatctacatggaggcagacgcatccgctcctcacgtcttcaaagccgatgaagcggctctcgttcccgcctacatcgaccaagatgccgtcctcaacgtctgccgcgagaagggcgtcacgatgattcacccgggctacggcttcctgagcgagaacgaggcgttcgcggccaaagtcgagaaagccgggatcatctggctcggaccgacgccgtcgcaaatcgaggcgatgggtctcaagcacgaagcgcgtgctcgtgcgatcaaggcggacgtccctgtccttccgggctcggaactcgtcgagacgctcgacttggcgctcgagcaggcgagcaaggtcggctacccgatcctcctgaaggcgactgcgggtggtggagggatgggaatgagcatctgcgggagcgagggcgagctcaagaaggcgttccagggcacgaccgacctcagcaaggtgcgtcttcgcttccttttgttcaactatctcaagctcacgctctcttccgcgcagaacctcttctc**catg**

>P*_LDP1_* (-362 ~ +3 of *LDP1* gene)

cacgcctctgtgactcggtacggagagagagagcgttgggttgggttggggattgtggcgagcgaagggcgacccagcagccagggagagggagttggtctggatgcaaaccatgcgcatctcctctcgcagttgaatcgtttttcccgctctgccctcgctctctcttccttctgctctttactcgctcacgaacaacaacgagccacacagcgtgagcacacaccgctgcactcactcgctgtcacggaccgcagctcacccttatcgtcactccctctcccaccgcacagcctcactccctctctcgctctccctcacaagcacaacacacggcacactcgcacgcacactcgcacgc**c**atg

>P*_LDP1in_* (-362 ~ +155 of *LDP1* gene)

cacgcctctgtgactcggtacggagagagagagcgttgggttgggttggggattgtggcgagcgaagggcgacccagcagccagggagagggagttggtctggatgcaaaccatgcgcatctcctctcgcagttgaatcgtttttcccgctctgccctcgctctctcttccttctgctctttactcgctcacgaacaacaacgagccacacagcgtgagcacacaccgctgcactcactcgctgtcacggaccgcagctcacccttatcgtcactccctctcccaccgcacagcctcactccctctctcgctctccctcacaagcacaacacacggcacactcgcacgcacactcgcacgcaatggccaccgtcaacgagaagcagcccgccaccgacgcgcccctcgcgcacgagaccgccatccaccgcgtgcgtccccatccctcccactgtcttcctcgtgaaacccgctcacccgttcgcaagcacacacgcaggtgtcggactacccc**atg**
