# Supplementary material for: Developing a set of strong intronic promoters for robust metabolic engineering in oleaginous Rhodotorula (Rhodosporidium) yeast species
Source: Microb Cell Fact. 2016 Nov 25;15:200. doi: 10.1186/s12934-016-0600-x (PMC5124236; doi:10.1186/s12934-016-0600-x)
Supplement: Supplementary file 2 — Additional file 2. Comparison of intronic and intronless GPD1 promoter strength. Luciferase gene assay was performed using the reporter strain with 795 and 932 bp promoter of GPD1 (−795 to +1) and GPD1in (−795 to +137), respectively. Cells were cultured in MinRL3 medium for 4 days and luciferase reporter assay was performed daily. [file 12934_2016_600_MOESM2_ESM.docx]

**
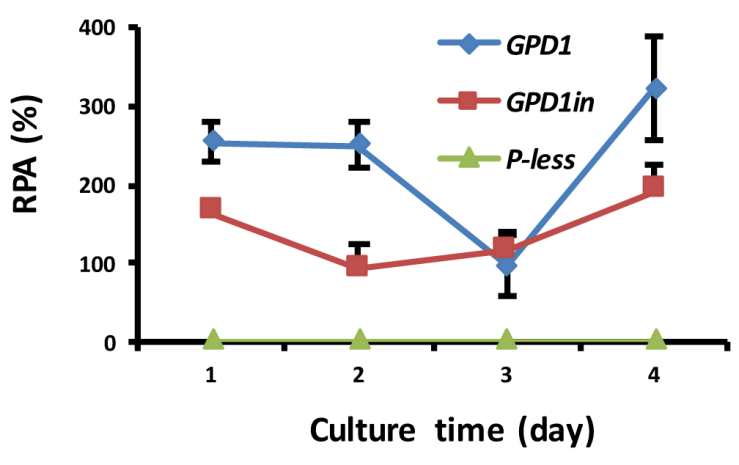
**

**Additional file 3. Comparison of intron-containing and intron-less *GPD1* promoter strength.** Luciferase assay was performed using the reporter strain with 795 bp and 932 bp promoter of *GPD1* (-795~+1) and *GPD1in* (-795~+137), respectively. Cells were cultured in MinRL3 medium for 4 days and luciferase reporter assay was performed daily.
